# CT assessment of right heart anatomy across tricuspid regurgitation severity grades: implications for transcatheter interventions

**DOI:** 10.1007/s10554-025-03505-8

**Published:** 2025-09-03

**Authors:** Domenico Angellotti, Polydoros Ν. Kampaktsis, Alessandro Sticchi, Hari Vivekanantham, Joanna Bartkowiak, Anna Franzone, Thomas Pilgrim, Nicolas Brugger, Stephan Windecker, Christoph Gräni, Susheel K. Kodali, Rebecca T. Hahn, Omar Khalique, Fabien Praz

**Affiliations:** 1 Department of Cardiology, Bern University Hospital, University of Bern, Bern, Switzerland; 2 Department of Advanced Biomedical Sciences, University of Naples Federico II, Naples, Italy; 3 Department of Medicine, Division of Cardiology, Structural Heart and Valve Center, Columbia University Medical Center, New York, NY USA; 4 Structural and Congenital Center, Hackensack University Medical Center, Hackensack, NJ USA; 5 University of Pisa, Pisa, Italy; 6 Department of Cardiology, University and Hospital of Fribourg, Fribourg, Switzerland; 7 St Francis Hospital and Heart Center, Roslyn, NY USA

**Keywords:** Tricuspid regurgitation, Computed tomography, Transcatheter tricuspid intervention, Patient selection

## Abstract

**Purpose:**

A five-grade severity scheme has been introduced for echocardiographic grading of tricuspid regurgitation (TR). Although higher TR grades have been associated with worse prognosis, it is unknown whether they can help determining patient eligibility for transcatheter tricuspid valve interventions (TTVI) and correspond to different anatomical phenotypes. The aim of our study was to investigate the relationship between TR severity and tricuspid valve (TV) anatomy and determine the screening failure rate for TTVI.

**Methods:**

The anatomy of TV, right heart and venae cavae in patients with significant TR having undergone cardiac CT scan, was investigated using 3-mensio software and correlated with TR severity determined by echocardiography.

**Results:**

One hundred patients from two tertiary centers were retrospectively included into the present analysis. Nine patients had moderate, 40 severe, 22 massive and 29 torrential TR. Pre-screening eligibility assessment showed comparable screening failure rates among patients with severe, massive and torrential TR for the Cardioband (35.0% vs. 40.9% vs. 34.5%, *p* = 0.79), TricValve (50.0% vs. 63.6% vs. 55.1%, *p* = 0.27), EVOQUE (30.0% vs. 27.3% vs. 31.1%, respectively, *p* = 0.84), Cardiovalve (50.0% vs. 36.4% vs. 44.8%, *p* = 0.37) and LuX-Valve (20.0% vs. 27.3% vs. 24.1%, *p* = 0.26) systems. No significant differences in CT-derived tricuspid annulus area, perimeter and diameter were observed between patients with severe, massive and torrential TR, while right atrium (*p* < 0.001) and right ventricle length (*p* = 0.014) significantly increased with progressive TR severity.

**Conclusion:**

CT imaging evaluation suggests similar eligibility rates for current TTVI devices and comparable TV anatomical phenotypes between patients with severe, massive, and torrential TR.

**Graphical abstract:**

CT-based pre-screening assessment for transcatheter tricuspid valve interventions according to TR severity.

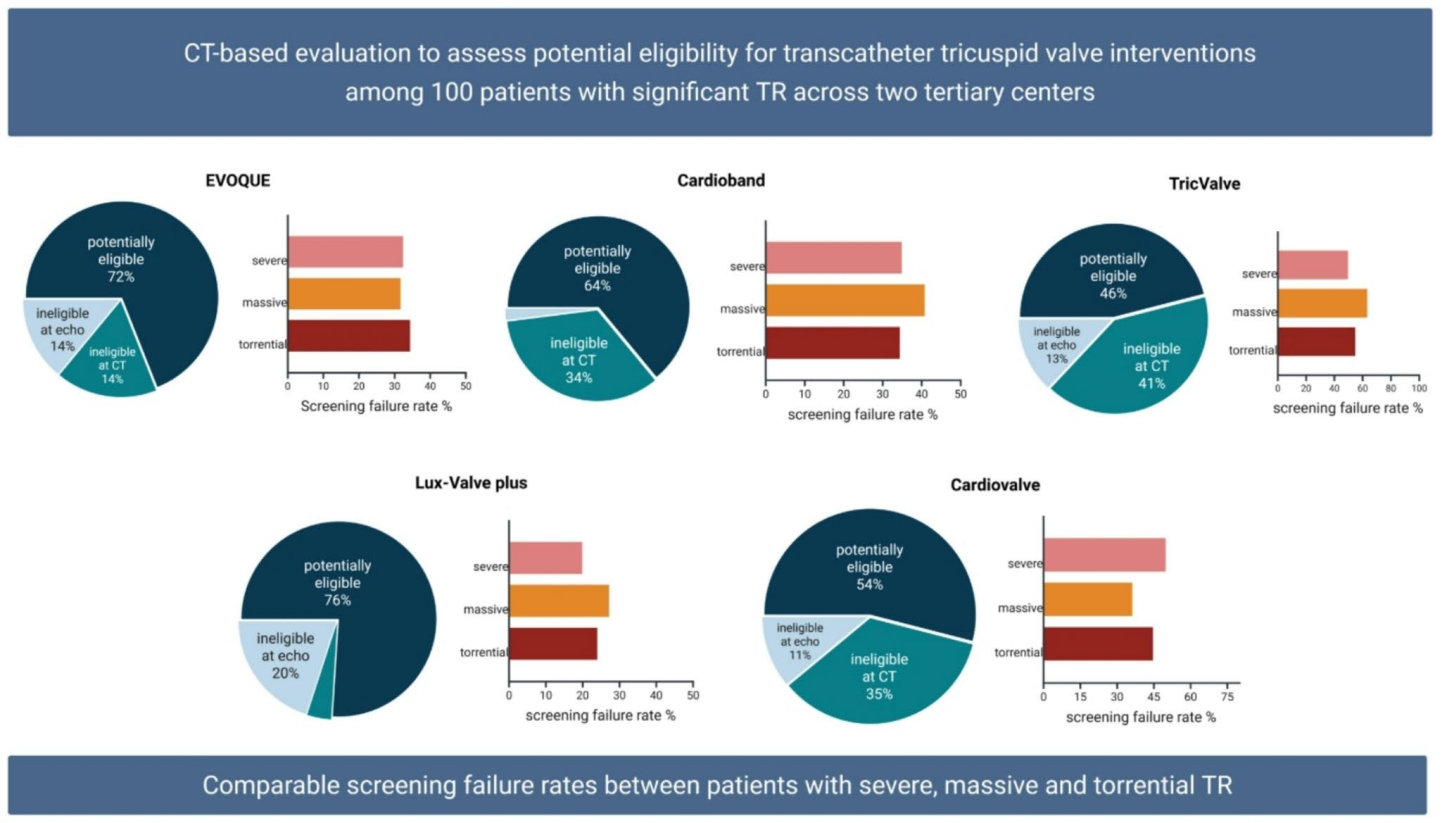

**Supplementary Information:**

The online version contains supplementary material available at 10.1007/s10554-025-03505-8.

## Introduction

Tricuspid regurgitation (TR) is a common and heterogeneous disease, independently associated with increased morbidity and mortality [1]. Among individuals aged 75 or older, approximately 4% develop at least moderate TR [2]. While TR rarely stems from intrinsic tricuspid valve (TV) leaflet abnormalities, the majority of cases arise from annular dilation or leaflet tethering due to enlargement of the right heart chambers. Pressure and volume overload due to TR leads to compensatory hypertrophy and dilatation of the right heart chambers [3]. Transthoracic (TTE) and transesophageal echocardiography (TEE) are the initial methods employed to diagnose and quantify TR. To provide a more nuanced classification of the broad TR spectrum, in particular in patients with advanced disease, a new grading scheme expanding to a 5-grade scale (mild, moderate, severe, massive, and torrential) was proposed. Studies have demonstrated the incremental prognostic value of the two additional grades (massive and torrential) in terms of mortality and rehospitalization for heart failure in patients with advanced disease [4]. However, the relationship between increasing TR severity and intrinsic anatomical changes, as well as the impact on treatment decisions have not been explored to date.

Transcatheter treatments are emerging solutions for patients unsuitable for surgery with, for the first time, a class IIb, level C recommendation in the 2021 European guidelines for the management of valvular heart disease [5]. While tricuspid transcatheter edge-to-edge repair represents a widely available treatment option, transcatheter direct annuloplasty and transcatheter replacement are commercially approved alternatives requiring careful screening with the use of cardiac computed tomography [6–10]. Notwithstanding, screening failure rates remain high (74%) as shown in the recently published TriACT registry [11].

The aim of the present study was to conduct a pre-screening evaluation of the right heart anatomy in patients with significant TR undergoing CT and to evaluate eligibility for current transcatheter tricuspid valve interventions (TTVI) other than edge-to-edge repair, including annuloplasty, orthotopic and heterotopic replacement devices.

## Methods

In this retrospective cohort study conducted at two tertiary centers (Bern University Hospital, Switzerland and Columbia University Medical Center, NYC, USA), we included patients with moderate or greater TR quantified by TTE and TEE, that had undergone an electrocardiogram-triggered CT scan with 0-100% RR interval scan for any indications. Patients with insufficient contrast enhancement of the right heart cavities were excluded. Patients enrolled in clinical trials for any of the devices considered were also excluded from the present analysis.

### Data collection

All baseline and clinical data were retrospectively recorded. TTE was performed by sonographers and evaluated by a board-certified cardiologist. TEE was performed by a board-certified cardiologist and evaluated by an independent second reader. For the ascertainment of echocardiographic parameters, measurements from at least three consecutive heartbeats were averaged. Echocardiographic loops were analyzed at a workstation allowing for offline analysis (Syngo Dynamics Workplace, version 9.5, Siemens Medical Solutions, Malvern, Pennsylvania). TR severity was graded using a combination of semi-quantitative and quantitative parameters, as described by the guidelines of the American Society of Echocardiography, as well as the European Association of Cardiovascular Imaging [12]– [13]. In addition, the extended grading system proposed by Hahn and Zamorano was applied to define ‘massive’ and ‘torrential’ TR [14]. The TV apparatus was imaged from parasternal inflow and the RV apical focus views on TTE and from midesophagus 4-chamber view on TEE. During TEE, biplanar and 3D images were acquired, with the latter being preferred when image quality was sufficient. Right ventricular (RV) dysfunction was defined as tricuspid annular plane systolic excursion < 17 mm and S’ < 9.5 cm/s.

**Fig. 1:**
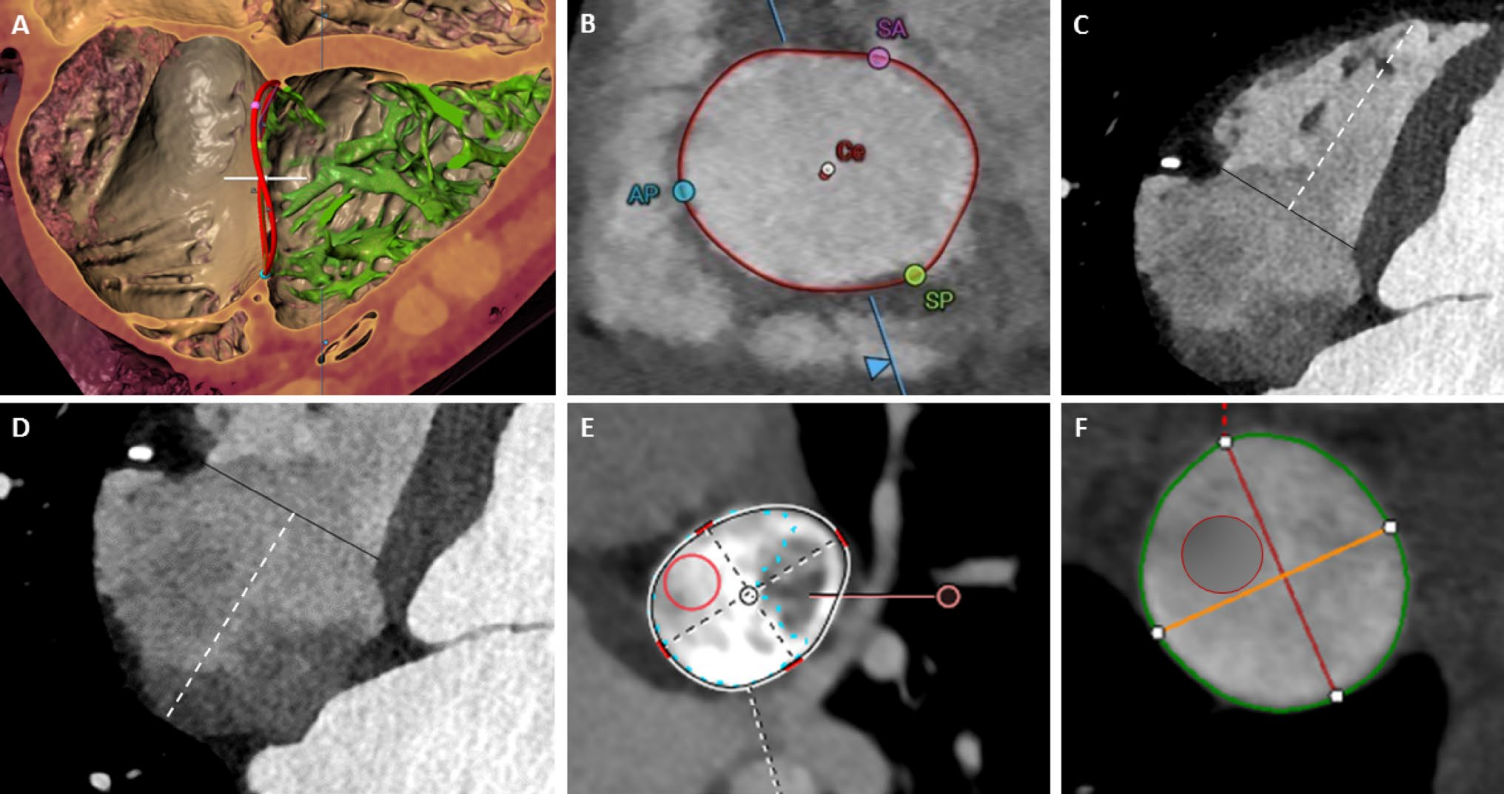
Computed tomography assessment of the tricuspid valve and right heart anatomy. After alignment of the multiplanar reformatting planes along the TV annulus and the RV apex in the orthogonal axial and coronal views, a reconstruction of the annulus (**A**) and planar cross-sectional area (short axis) of the annulus are obtained (**B**), allowing measurement of the maximal/minimal diameter, perimeter and area. 4CH view (**C**): Black line = Tricuspid annulus; Dashed white line = RV length. 4CH view (**D**): Black line = Tricuspid annulus; Dashed white line = Right atrium length. Superior vena cava diameters (**E**). Inferior vena cava diameters (**F**)

CT measurements were performed on a 0-100% RR interval scan with 5% increment reconstructions using a right heart-specific protocol. The mean Hounsfield Units (± standard deviation) were as follows: 461.4 ± 128.9 for RA analysis, 325.2 ± 122.8 for RV analysis, 587.4 ± 439.1 for SVC analysis, and 243.1 ± 140.2 for IVC analysis. A total of 37 variables including TV annulus area, perimeter and diameters, right atrium (RA) and RV length (dimension from middle of TV annulus to RA roof and RV apex, respectively), superior vena cava (SVC) and inferior vena cava (IVC) area, perimeter and diameters, as well as the distance between the right coronary artery (RCA) and the TV annulus, were analyzed during mid-diastole and repeated during systole to investigate dynamic changes during the cardiac cycle (Supplementary Material). The 3-Mensio structural heart software (tricuspid valve module, Pie Medical Imaging, Maastricht, Netherlands) was used for all measurements (Fig. 1). Imaging data and CT measurements were collected with readers blinded to clinical and echocardiographic data.

Theoretical TTVI eligibility assessment was conducted by two investigators (D.A. and F.P) and relied on previously reported clinical and anatomical considerations for each device [7–9, 15, 16]. The screening evaluation did not involve manufacturers and never included information from actual screening reports shared with the companies. Screening failure rates were determined for the following TTVI devices: the Cardioband tricuspid system (Ewards Lifescience, Irvine, California) for transcatheter TV repair; the TricValve^®^ bicaval valve system (P + F Products Features GmbH, Vienna, Austria) for transcatheter TV heterotopic replacement; the EVOQUE system (Edwards Lifescience, Irvine, California), the Cardiovalve system (Venus Medtech, Yahadut, Canada), and the LuX-Valve Plus system (Jenscare Biotechnology, Ningbo, China) for transcatheter TV orthotopic replacement; Anatomical and hemodynamic criteria assessed at echo (i) and CT anatomical criteria (ii) were taken into consideration. One value out of range was considered to indicate screening failure.

Patients were deemed suitable for Cardioband implantation in case of (i) secondary etiology of TR due to annulus dilatation and a sPAP ≤ 65 mmHg; moreover, the following CT inclusion criteria (ii) were adopted: TV annulus perimeter from aorta to coronary sinus between 73 and 120 mm and RCA proximity to the anchors > 6.90 mm and RA length > 89 mm. TricValve implantation was considered feasible in patients with (i) TAPSE > 13 mm, sPAP < 65 mmHg as assessed by TTE. For SVC valve implantation seven CT measurements (ii) were required, including diameter of SVC at level of top of Pulmonary Artery (19–34 mm) and at level of middle of Pulmonary Artery (22–34 mm). For implantation of the IVC valve five measurements were required, including IVC-RA transition diameter and IVC at top of Hepatic Veins (24–35 mm for both). Patients were considered eligible for the EVOQUE system in case of (i) tricuspid annular plane systolic excursion (TAPSE) > 13 mm, LVEF > 25% and sPAP < 70 mmHg at TTE and (ii) TV annulus projected perimeter at mid-diastole ranged between 114 mm and 169 mm at CT (corresponding to a perimeter derived diameter between 36.5 and 53.8 mm). Patients were considered borderline for EVOQUE implantation, if the CT projected perimeter ranged between 169 mm and 180.5 mm. For the present analysis, patients considered borderline were included among eligible subjects. Regarding the Cardiovalve system, patients were considered eligible if (i) TAPSE > 13 mm on TTE and (ii) TV annulus diameter at mid-diastole ranged between 45 and 60 mm on CT. Patients were considered eligible for LuX-Valve in case of (i) sPAP < 55 mmHg and TAPSE > 10 mm and (ii) TV annulus diameter at mid-diastole between 35 and 75 mm at CT. A detailed description of the CT criteria used for each device is shown in Supplementary Material.

Patient’s identifying information have been omitted. A waiver was obtained from each ethics committee to retrospectively analyzed the imaging data of the included patients. The study conformed to the Declaration of Helsinki on human research and was approved by the local ethics committee.

### Statistical analysis

Normality of distributions for continuous variables was tested using the Shapiro-Wilk test. Continuous variables are expressed as mean ± standard deviation, and were compared by ANOVA test. Categorical variables are reported as counts and percentages and were compared using the Chi-square test for independent variables and Wilcoxon signed-rank test for paired variables. Pearson correlation coefficients and linear regression were used to assess the correlations between CT measurements and TTE parameters. Intraclass Correlation Coefficient test was used to assess interobserver variability of the CT measurements. Statistical significance was assumed for *p* < 0.05. Statistical analysis was performed using IBM SPSS version 25 (IBM, Armonk, NY, USA).

## Results

### Clinical and echocardiographic characteristics

**Fig. 2:**
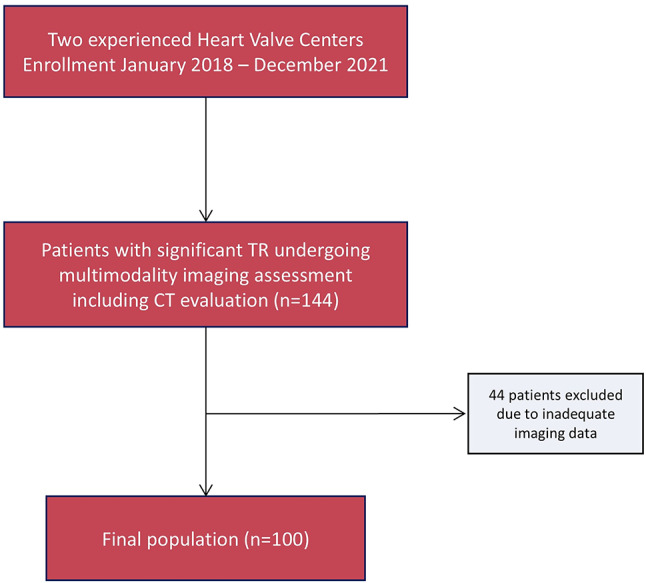
Study flow-chart

Between January 2018 and December 2021, 144 patients with moderate or greater TR as assessed by echocardiography underwent an electrocardiogram-triggered cardiac CT scan. Of these, 44 were excluded due to inadequate imaging quality (e.g., insufficient contrast enhancement of the right heart chambers), leaving 100 patients who were retrospectively included in the final study population (Fig. 2). Baseline characteristics and echocardiographic data are displayed in Table 1. Nine patients (9%) had moderate, 40 severe (40%), 22 massive (22%) and 29 torrential TR (29%). The mean age was 78.9 ± 10.0 years and 56.0% of patients were women. A total of 80 patients (80%) had atrial fibrillation while 38 (38%) had previous left-sided valve surgery. An RV pacemaker lead was present in 34 patients (34%) and in 12 of them, it contributed to TR. Comorbidities, including previous coronary artery disease and chronic kidney disease, were highly prevalent, with no differences between TR grades. The mechanism of TR was secondary in 84.0% of the patients, with 53 (53%) having atrial functional and 31 (31%) ventricular functional TR. The mean vena contracta (VC) width of the tricuspid jet was 1.1 ± 0.55 cm, overall. The effective regurgitant orifice area measured using the PISA method was 0.67 ± 0.51 and regurgitant volume was 83.0 ± 42.1 ml. All three semi-quantitative or quantitative parameters progressively increased with increasing TR grades. The TV annulus was dilated in most of patients with a major diameter of 27.5 ± 6.5 mm/m^2^ (indexed to body surface area). The mean TV coaptation depth was 11.9 ± 5 mm. The regurgitant jet was central in 76.9% of the cases. RV dysfunction was present in 56.3% of the patients, with no differences among TR grades. The inferior vena cava was enlarged in 60.5% of patients, with a mean diameter during inspiration of 24.6 ± 6 mm. The left ventricular ejection fraction was generally preserved, with a mean of 51.6 ± 14.2% and did not differ across TR grades; significant mitral regurgitation was present in 27% of patients, with nine of them having severe MR.

**Table 1:** Baseline clinical and echocardiographic characteristics

	Overall (100)	Moderate TR (9)	Severe TR (40)	Massive TR (22)	Torrential TR (29)	*p* value among TR grades
*Comorbidities*						
Age	78.9 ± 10.0	86.9 ± 14.2	77.5 ± 9.1	78.6 ± 11.3	81.9 ± 10.0	0.061
Female	56 (56.0%)	9 (100%)	18 45.0%)	14 (63.6%)	15 (51.7%)	0.021
BMI (kg/m^2^)	25.0 ± 4.6	28.2 ± 5.1	25.3 ± 4.8	23.7 ± 3.5	24.5 ± 4.4	0.091
CAD	44 (44.0%)	4 (44.4%)	18 (45.0%)	8 (36.4%)	14 (48.3%)	0.86
Previous AVR	20 (20.0%)	2 (22.2%)	9 (22.5%)	3 (13.6%)	6 (20.7%)	0.86
Previous MVR/repair	18 (18.0%)	2 (22.2%)	5 (12.5%)	4 (18.2%)	4 (24.1%)	0.64
Previous TAVI	14 (14.0%)	3 (33.3%)	3 (7.5%)	3 (13.6%)	5 (17.2%)	0.21
Previous M-TEER	5 (5.0%)	1 (11.1%)	(5.0%)	0 (0.0%)	2 (6.9%)	0.57
eGFR < 30 ml/min	38 (38.0%)	3 (33.3%)	19 (47.5%)	6 (27.2%)	10 (34.5%)	0.43
Atrial fibrillation	80 (80.0%)	7 (77.8%)	29 (72.5%)	20 (90.9%)	24 (82.7%)	0.16
RV lead	34 (34.0%)	1 (11.1%)	14 (35.0%)	9 (40.9%)	10 (34.5%)	0.46
*Echocardiography*						
TR etiology						0.42
primary	7 (7.0%)	1 (11.1%)	3 (7.5%)	2 (9.1%)	1 (3.4%)	
Secondary *Atrial* *Ventricular*	84 (84%) *53 (53%)* *31 (31%)*	8 (88.9%) *6 (66%)* *2 (22%)*	33 (82.5%) *21 (52%)* *12 (30%)*	20 (91.0%) *10 (45.4%)* *10 (45%)*	23 (79.3%) *16 (55%)* *7 (24%)*	
mixed	9 (9.0%)	0 (0.0%)	4 (10.0%)	0 (0.0%)	5 (5.0%)	
TV annulus, mm (parasternal view)	45.7 ± 6.7	44.8 ± 5.4	45.8 ± 6.7	46.1 ± 7.2	46.8 ± 5.9	0.37
TR EROA by PISA (mm^2^)	0.67 ± 0.51	0.38 ± 0.17	0.56 ± 0.20	0.82 ± 0.75	1.24 ± 0.84	0.003
TR RVol by PISA (ml)	83.0 ± 42.1	60.1 ± 13.3	68.0 ± 25.1	78.6 ± 43.2	113.3 ± 49.1	0.004
TR Vena contracta	11.8 ± 5.6	5.5 ± 0.7	9.5 ± 1.9	12.1 ± 4.4	17.3 ± 7.1	< 0.001
MR ≥ 2+	41 (41.0%)	6 (66.7%)	22 (55.0%)	7 (31.8%)	6 (20.7%)	0.15
LVEF%	51.6 ± 14.2	55.0 ± 11.9	49.7 ± 14.6	50.7 ± 14.4	54.3 ± 14.9	0.58
RV basal diameter (mm)	48.8 ± 1.0	37.8 ± 0.8	49.3 ± 1.4	49.0 ± 2.5	51.3 ± 1.9	0.008
TAPSE, mm	14.5 ± 5.1	17.0 ± 6.8	13.7 ± 5.5	14.7 ± 5.4	15.4 ± 5.2	0.12
RV S-TDI, cm/s	8.6 ± 2.9	8.1 ± 2.8	8.5 ± 3.1	8.5 ± 2.7	10.2 ± 2.9	0.54

*BMI*: body mass index; *AVR*: aortic valve replacement; *TAVI*: transcatheter aortic valve implantation; *VR*: mitral valve replacement; *M-TEER*: mitral transcatheter edge-to-edge repair; *eGFR*: Estimated Glomerular Filtration Rate; *COPD*: Chronic obstructive pulmonary disease *PPM*: permanent pacemaker; *MR*: mitral regurgitation; *LVEF*: left ventricle ejection fraction; *RV*: right ventricle; *PHT*: Pressure Half Time; *sPAP*: systolic pulmonary artery pressure; *EROA*: effective regurgitant orifice area; *PISA*: Proximal Isovelocity Surface Area; *RVol*: regurgitant volume; *TR*: tricuspid regurgitation

### Assessment of TTVI eligibility

**Fig. 3:**
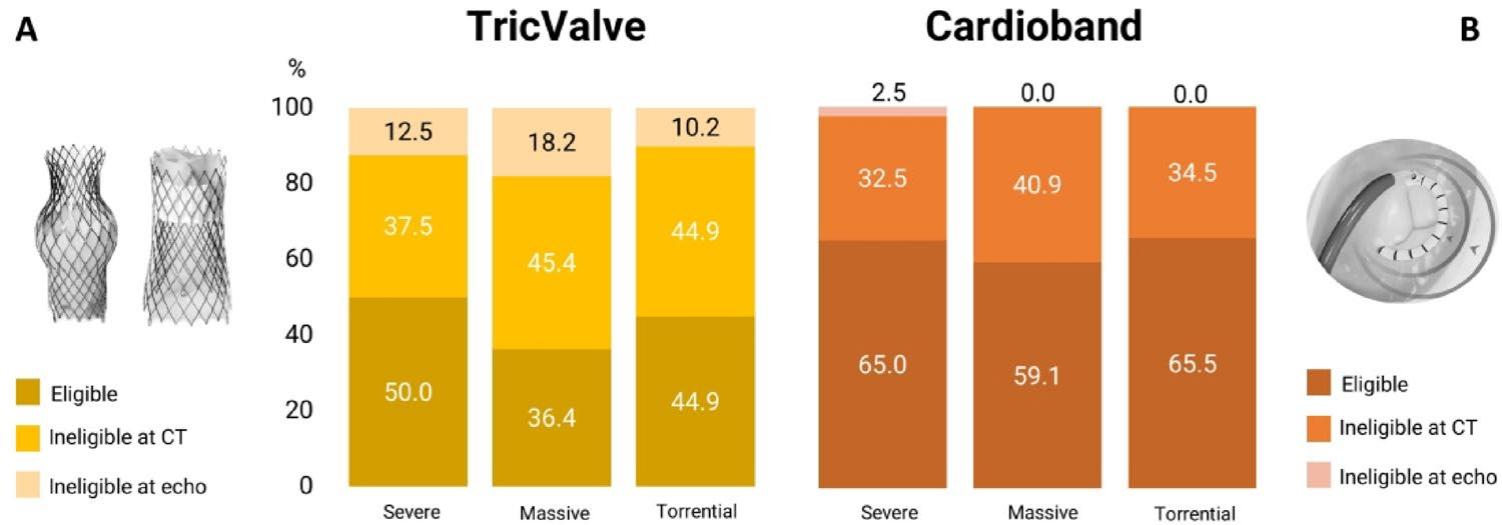
Eligibility assessment for TricValve and Cardioband systems according to TR severity

The rate of eligibility for Cardioband implantation was 64% overall, without difference in patients with severe compared to those with massive and torrential TR (screening failure rate 35.0% vs. 40.9% vs. 34.5%, *p* = 0.79). 34/36 subjects (94.5%) were excluded due to anatomical considerations. Of these, twelve patients had a reduced distance of the RCA from the anchors while 22 were excluded due to TV annulus dimensions. Two patients (5.5%) were deemed ineligible due to severe pulmonary hypertension. The TricValve Transcatheter Bicaval Valves System implantation was considered feasible in 46 patients (46%). 41/56 patients met CT anatomical exclusion criteria (IVC diameter larger than 35 mm) while 15 were deemed ineligible due to severe RV dysfunction at TTE. Patients with severe TR did not show a higher screening failure rate compared to those with massive and torrential TR (50.0% vs. 63.6% vs. 55.1%, *p* = 0.27) (Fig. 3).

Considering both echocardiographic and CT anatomical criteria, 72% of patients were deemed eligible for EVOQUE implantation. 28 patients (28%) were considered ineligible: 14 due to CT anatomical criteria (annulus too large) and 14 due to echocardiographic derived parameters (9 due to severe pulmonary hypertension, 5 due to severe RV dysfunction). Patients with severe TR did not show higher screening failure rate compared to patients with massive TR as well as those with torrential TR (30.0% vs. 27.3% vs. 31.1%, respectively, *p* = 0.84). In 13 patients, EVOQUE implantation was considered borderline, without differences between groups.

A total of 54 patients (54%) were considered eligible for Cardiovalve implantation without difference between severe, massive and torrential TR patients (screening failure 20/40, 50.0% vs. 8/22, 36.4% vs. 44.8%, *p* = 0.37). 35/46 (76.1%) of subjects were excluded due to TV annulus too large at CT, while 11 (23.9%) patients were deemed ineligible due to severe RV dysfunction and severe pulmonary hypertension according to TTE (4 and 7 patients, respectively).

**Fig. 4:**
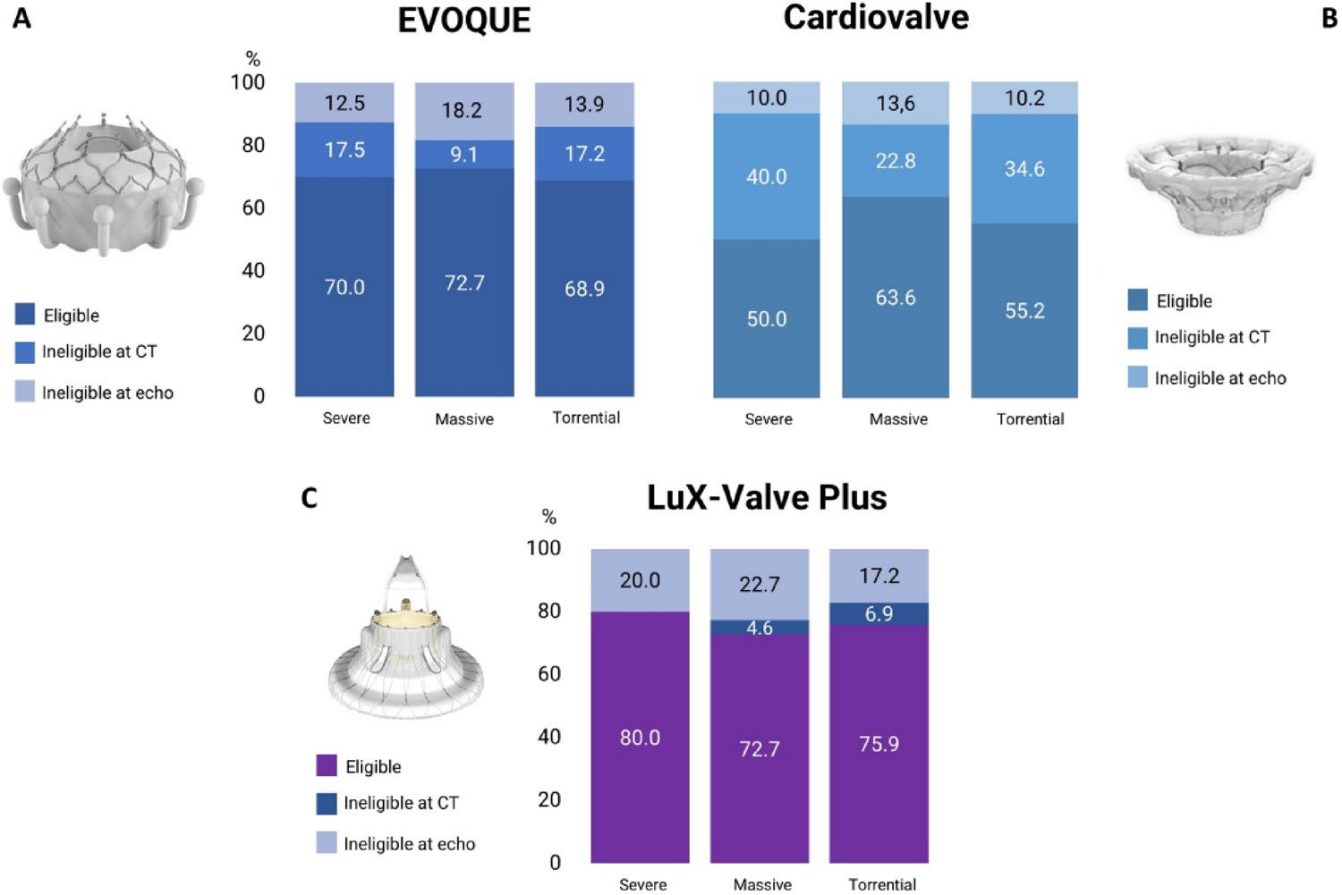
Eligibility assessment for EVOQUE, Cardiovalve and LuX-Valve Plus systems according to TR severity

A total of 76 patients (76%) were deemed eligible for LuX-Valve plus system (4/24 were anatomically ineligible and 20 were excluded due to clinical reasons). Patients with severe TR did not show higher screening failure compared to those with massive TR as well as those with torrential TR (20.0% vs. 27.3% vs. 24.1, *p* = 0.26). Of the 24 patients deemed ineligible for the procedure, 15 were excluded due to severe pulmonary hypertension and 9 due to severe RV dysfunction at TTE (Fig. 4).

### CT anatomy

**Fig. 5:**
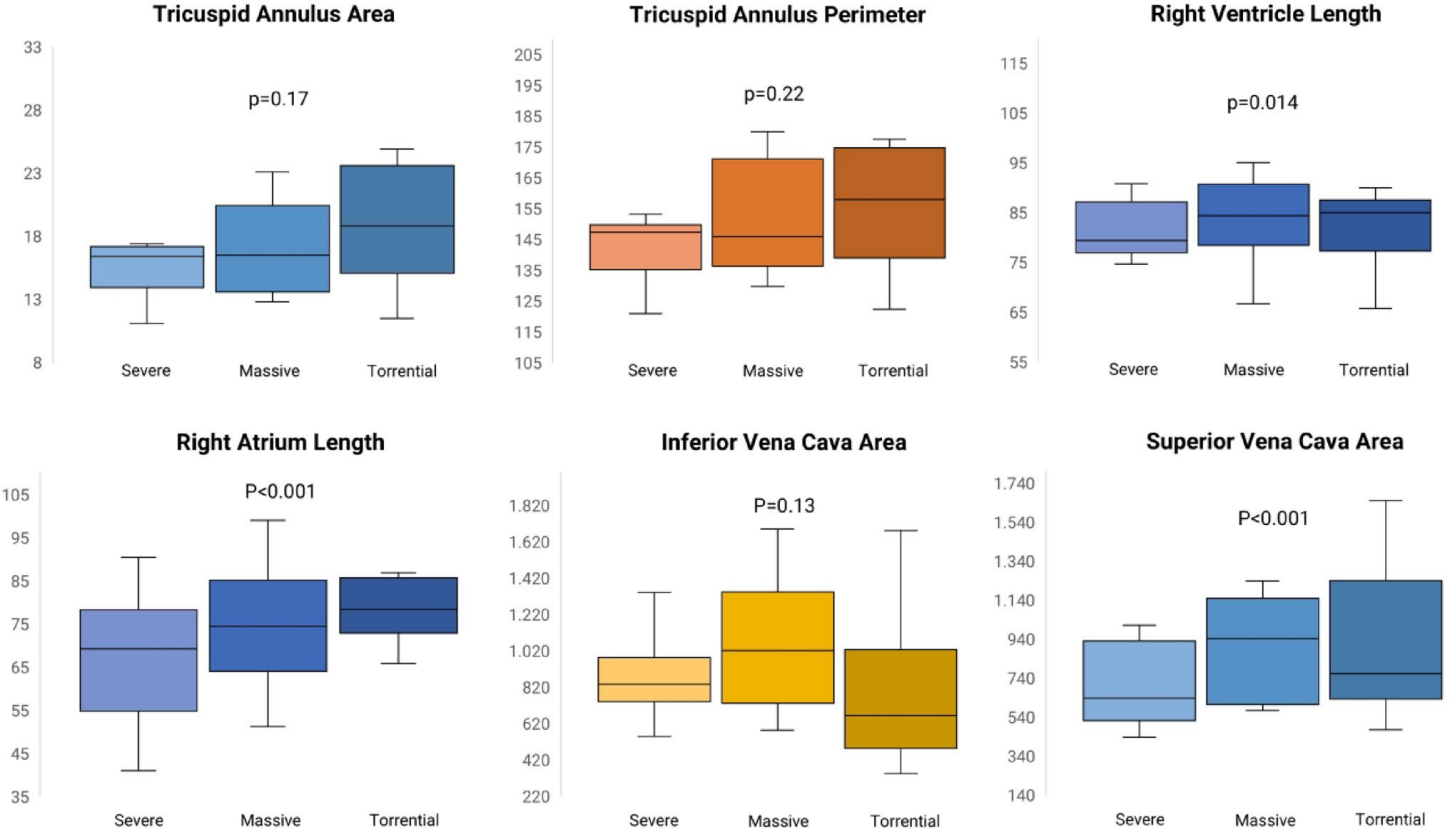
Computed tomography measurements according to TR severity

Differences in main CT parameters across TR severe grades are summarized in Table 2; Fig. 5. TV annulus area in mid-diastole was 17.6 ± 5.0  cm^2^ overall; 13.3 ± 1.6 cm^2^ for moderate TR, 17.3 ± 4.3 cm^2^ for severe TR, 18.0 ± 5.7 cm^2^ for massive TR and 18.8 ± 5.1  cm^2^ for torrential TR. There was no significant difference in TV annulus area between patients with severe, massive and torrential TR (*p* = 0.17). TV annulus perimeter at mid-diastole showed a similar pattern with a mean for the whole cohort of 149.4 ± 24.0 mm; 135.6 ± 11.1 mm for moderate TR, 153.4 ± 20.6 mm for severe TR, 158.8 ± 25.9 mm for massive TR, and 164.6 ± 20.4 mm for torrential TR, without significant differences within the severe grades of the disease (*p* = 0.22). The inferior vena cava (IVC) ostium area was 882.7 ± 319.4 mm^2^ overall; 638.3 ± 227.8 mm^2^ for moderate TR, 828.9 ± 298.1 mm^2^ for severe TR, 1025.7 ± 463.1 mm^2^ for massive TR, 943.8 ± 380.8 mm^2^ for torrential TR. Similarly, the difference was not significant when comparing patients the severe grades of TR (*p* = 0.13). Conversely, right atrium length (67.2 ± 13.4 mm vs. 71.7 ± 11.8 mm vs. 81.5 ± 13.2 mm, *p* < 0.001) and RV length (77.9 ± 9.2 vs. 81.7 ± 12.0 mm vs. 84.5 ± 10.6 mm, *p* = 0.014) significantly increased between patients with severe, massive and torrential TR.

**Table 2:** Computed tomography assessment of right heart anatomy according to TR severity (diastole)

	Overall (100)	Severe TR (40)	Massive TR (22)	Torrential TR (29)	*p*-value*	Van Rosendael et al. (25) ≥3 + TR
Annulus Area (cm^2^)	17.6 ± 5.0	17.3 ± 4.3	18.0 ± 5.7	18.8 ± 5.1	0.17	16.1 ± 2.9
Annulus perimeter (mm)	149.4 ± 24.0	153.4 ± 20.6	158.8 ± 25.9	164.6 ± 20.4	0.22	148.4 ± 15.5
Perimeter derived Annulus diameter (mm)	47.0 ± 7.8	47.1 ± 6.7	47.2 ± 7.7	48.4 ± 7.2	0.13	-
4ch max RA length (mm)	71.8 ± 14.3	67.2 ± 13.4	71.7 ± 11.8	81.5 ± 13.2	< 0.001	-
4ch max RV length (mm)	80.5 ± 10.8	77.9 ± 9.2	81.7 ± 12.0	84.5 ± 10.6	0.014	-
SVC ostium area (mm^2^)	788.4 ± 395.5	679.4 ± 285.9	784.6 ± 278.2	1001.8 ± 434.4	< 0.001	-
IVC ostium area (mm^2^)	882.7 ± 319.4	828.9 ± 298.1	1025.7 ± 463.1	943. ± 380.8	0.13	674 ± 193

*TR*: tricuspid regurgitation; *RA*: right atrium; *RV*: right ventricle; 4-ch-four-chamber view; *SVC*: superior vena cava; *IVC*: inferior vena cava. *ANOVA test p-value between severe, massive and torrential TR patients

### Changes during cardiac cycle

The differences in CT parameters between diastolic and systolic measurements are shown in Table 3. Paired analyses showed a significant variability between TV annulus area measured in systole vs. diastole in patients with moderate TR (12.3 ± 1.7 vs. 13.3 ± 1.6 cm^2^ *p* = 0.020), as well as in those with severe TR (15.2 ± 4.1 vs. 16.9 ± 4.3 cm^2^ *p* < 0.001), but to a lesser extent in those with massive (16.9 ± 5.9 vs. 18.0 ± 5.7 cm^2^ *p* = 0.054) or torrential TR (18.8 ± 5.1 vs. 19.5 ± 4.7 cm^2^ *p* = 0.075). TV annulus perimeter and perimeter-derived diameter showed a similar pattern with significant variability during cardiac cycle only in the patients with moderate and severe TR. Similarly, the IVC ostium area changed significantly when comparing systolic and diastolic measurements in patients with moderate TR (623.6 ± 246.6 vs. 638.3 ± 227.8 mm^2^ *p* < 0.001) as well as severe TR (810.4 ± 298.1 vs. 828.9 ± 298.1 mm^2^ *p* < 0.001), but not in patients with massive TR (946.9 ± 300.2 vs. 1025.7 ± 463.1 mm^2^ *p* = 0.169), or torrential TR (892.7 ± 371.0 vs. 943.8 ± 380.8 mm^2^ *p* = 0.283). A similar pattern was observed when analyzing SVC area changes during cardiac cycle. In contrast, the right atrium (larger during systole) and right ventricle length (smaller during systole) had consistent variability between systole and diastole across the severe grades of TR.

**Table 3:** Computed tomography assessment of right heart anatomy in systole and diastole

	Moderate TR *n* = 9			Severe TR *n* = 40			Massive TR *n* = 22			Torrential TR *n* = 29		
	S	D	*P* value	S	D	*P* value	S	D	*P* value	S	D	*P* value
Annulus Area (cm^2^)	12.3 ± 1.7	13.3 ± 1.6	0.020	15.2 ± 4.1	16.9 ± 4.3	< 0.001	16.9 ± 5.9	18.0 ± 5.7	0.054	18.8 ± 5.1	19.5 ± 4.7	0.075
Annulus perimeter (mm)	131.2 ± 10.5	135.6 ± 11.1	0.006	144.7 ± 18.9	153.4 ± 20.6	< 0.001	154.5 ± 24.7	158.8 ± 25.9	0.058	160.9 ± 22.4	164.6 ± 20.4	0.064
Perimeter derived annulus diameter (mm)	37.2 ± 2.7	39.5 ± 3.5	0.045	43.7 ± 6.9	47.1 ± 6.7	< 0.001	45.7 ± 8.6	47.2 ± 7.7	0.071	47.6 ± 8.9	48.4 ± 7.2	0.128
4ch max RA length (mm)	63.1 ± 7.9	58.5 ± 6.8	0.006	71.4 ± 14.6	67.2 ± 13.4	< 0.001	74.6 ± 11.8	71.7 ± 11.8	< 0.001	84.1 ± 13.1	81.5 ± 13.2	< 0.001
4ch max RV length (mm)	62.5 ± 13.4	76.8 ± 10.4	0.238	68.3 ± 11.3	77.9 ± 9.2	< 0.001	71.0 ± 12.4	81.7 ± 12.0	< 0.001	75.4 ± 12.3	84.5 ± 10.6	< 0.001
SVC ostium area (mm2)	521.9 ± 109.1	488.7 ± 138.6	< 0.001	745.0 ± 313.9	679.4 ± 285.9	< 0.001	808.9 ± 304.6	784.6 ± 278.2	0.065	1001.8 ± 434.4	1012.9 ± 512.6	0.898
IVC ostium area (mm2)	623.6 ± 246.6	638.3 ± 227.8	0.001	810.4 ± 298.1	828.9 ± 298.1	< 0.001	946.9 ± 300.2	1025.7 ± 463.1	0.169	892.7 ± 371.0	943.8 ± 380.8	0.283

*S*: systole; *D*: diastole; *TR*: tricuspid regurgitation; *RA*: right atrium; *RV*: right ventricle; 4-ch-four-chamber view; *SVC*: superior vena cava; *IVC*: inferior vena cava

### Correlations between echocardiographic and CT parameters and reproducibility

The correlations between the different CT anatomical parameters, as well as echocardiography derived regurgitant volume and VC are displayed in Figure S1 and Table S1. The intraclass correlation coefficient showed a very high reproducibility of CT measurements with a level of agreement of 0.949 for the TV annulus area measurements between two blinded examiners on a sample of 10 patients (*Table S2*).

## Discussion

The main findings of our study are the following:

1. Anatomical screening failure rates for current TV annuloplasty and replacement devices were comparable among patients with severe, massive, or torrential TR (Central Illustration).
2. Although TV annular dimensions steadily increase across TR grades, no statistically significant differences were found between patients with severe and those with massive or torrential TR.
3. Annular variability during the cardiac cycle was preserved in patients with moderate and severe TR, while statistical significance was not reached in patients with massive and torrential TR.

The proposed extended 5-grade scheme has been shown to correlate with mortality, presumably due to increasing TR severity that has been closely linked to outcome even beyond the generally accepted effective regurgitant orifice area (EROA) cut-off of 40 mm [2] [17]– [18]. However, the variations in right heart remodeling across these different severity grades, as well as its influence on TTVI eligibility have not been investigated to date.

The present study undertook a comprehensive evaluation of the TV and right heart anatomy among a population with significant TR representing the spectrum of patients referred for treatment in daily clinical practice. Baseline characteristics were well aligned with other series including majority of female patients (56%), with frequent history of long-standing atrial fibrillation (80%) and renal failure (38%), as well as the presence of a preexisting RV lead in about one third of the patients [1, 19].

Assessment of eligibilty for transcatheter tricuspid valve replacement is mainly based on annular dimensions, while the distance to the RCA represents an additional factor considered to determine the eligibility for direct annuloplasty with the Cardioband system [20, 21]. In our study, screening failure rates for TTVI were similar among patients with severe, massive and torrential TR, when considering anatomical and clinical suitability for both tricuspid annuloplasty and replacement (either orthotopic or heterotopic). These findings suggest a predominant role of anatomical phenotypes over TR severity impacting patient selection.

CT-derived TV annular dimensions were comparable between patients with severe and those with massive or torrential TR. This observation supports the known heterogeneity of the patients presenting with symptomatic TR. RA volume is the main determinant of TV annulus size [21]and TV annulus dilatation may differ by the presence or absence of atrial fibrillation [22]The ratio of RA to RV area is one of the main parameters which may distinguish between atrial (ratio ≥ 1.5) and ventricular (ratio < 1.5) subgroups of secondary TR [23, 24]. Indeed, continuous annulus dilatation may not extend indefinitely and other mechanisms like leaflet tethering, potentially accentuated by papillary muscle displacement or as a consequence of pressure overload or dyssynchrony, as well as the presence of RV lead may contribute to TR progression without further TV annulus. This may also translate into the development of a ‘shelf anatomy’ secondary to annular ingrowth that has been specifically described in patients with atrial secondary TR [25, 26].

Moreover, the reduced variability in annular size during the cardiac cycle observed in patients with more-than-severe TR supports the concept of a ‘fixed’ annular anatomy. This concept is further supported by similar observations concerning the caval dimensions, despite substantial volume overload in massive and torrential TR. As with TV annulus dimensions, IVC size is influenced by multiple factors including RA pressures (and not just TR volume), age and body size [27].

Similar values were obtained in 40 patients with TR ≥ 3 + in a previous study in terms of annular dimensions [28]. Specifically, the authors reported a mean TV annulus area of 16.1 ± 2.9 cm² and a perimeter of 148.4 ± 15.5 mm for subjects with moderate or severe TR. In our cohort of 40 patients with severe TR, the mean TV annulus area and perimeter were 17.3 ± 4.3 cm² and 153.4 ± 20.6 mm, indicating a comparable patient population (Table 2). However, the paper by van Rosendael et al. was published before the new five-grade TR classification became widely adopted and did therefore not address the anatomical phenotypes of higher TR severity.

The present study introduces the concept of an “anatomic ceiling” as right heart and tricuspid annulus dilatation may not extend indefinitely as TR progresses, potentially explaining the absence of a definitive correlation between screening failure rates and TR severity, particularly for non-TEER devices. Moreover, it confirms the multifactorial nature of TR progression, which is determined not only by the size of the TV annulus but also by alterations in leaflet coaptation, influenced by a complex interplay of right heart remodeling, leaflet tethering, leaflet growth, and pulmonary pressures [29]. Consistent with this, the prevalence of atrial secondary etiology (primarily driven by annular dilatation) did not differ significantly across the severe TR grades, whereas RV basal diameter increased significantly with worsening TR.

### Limitations

Our findings should be interpreted in view of the following limitations: (a) the relatively small sample size resulted in the identification of small patient subgroups, which may have limited the study’s power to detect statistically significant differences and increased the risk of a type I error. In this context, the absence of significant differences in TV annulus measurements across TR grades (despite the numerical increase with increasing TR severity) should be interpreted with caution; moreover, a significant proportion of patients (44/144) were excluded from the analysis due to inadequate imaging quality and this may introduce a selection bias; (b) Imaging data were site reported, without a corelab for images re-evaluation; (c) TR is dynamic and the variability in right heart dimensions based on individual loading conditions at the time of CT scan should be considered; moreover, pericardial constraint and the RV-LV interaction, may drive the relatively reduced ability of the annulus to both continue to dilate, as well as exhibit normal cyclic variability. (d) screening failure rates need to be considered with caution, as eligibility assessment is based on theoretical assumptions not shared with manufacturers, while treatment decisions in clinical practice are taken individually, based on the integration of several parameters. The presence of concomitant significant MR in a considerable proportion of patients (27%) could also impact patient management and physician decision. Moreover, algorithms for screening decisions constantly change over time as we keep learning more about the device, and new device sizes are available.

## Conclusions

CT-based pre-screening assessment suggests similar eligibility rates for current TTVI devices and comparable TV anatomical phenotypes between patients with severe TR and those with massive and torrential TR.

## Supplementary Information

Below is the link to the electronic supplementary material.

Supplementary Material 1

## Data Availability

All data will be provided by the corresponding author upon a reasonable request.

## References

[1] Topilsky Y, Maltais S, Medina Inojosa J, Oguz D, Michelena H, Maalouf J et al (2019) Burden of tricuspid regurgitation in patients diagnosed in the community setting. JACC Cardiovasc Imaging 12:433–442 30121261 10.1016/j.jcmg.2018.06.014

[2] Coffey S, Roberts-Thomson R, Brown A, Carapetis J, Chen M, Enriquez-Sarano M et al (2021) Global epidemiology of valvular heart disease. Nat Rev Cardiol 18:853–864 34172950 10.1038/s41569-021-00570-z

[3] Praz F, Beyersdorf F, Haugaa K, Prendergast B (2024) Valvular heart disease: from mechanisms to management. Lancet 403:1576–1589 38554728 10.1016/S0140-6736(23)02755-1

[4] Hahn RT, Thomas JD, Khalique OK, Cavalcante JL, Praz F, Zoghbi WA (2019) Imaging assessment of tricuspid regurgitation severity. JACC Cardiovasc Imaging 12:469–490 30846122 10.1016/j.jcmg.2018.07.033

[5] Vahanian A, Beyersdorf F, Praz F, Milojevic M, Baldus S, Bauersachs J et al (2022) 2021 ESC/EACTS guidelines for the management of valvular heart disease. Eur Heart J 43:561–632 34453165 10.1093/eurheartj/ehab395

[6] Lurz P, Besler C, Schmitz T, Bekeredjian R, Nickenig G, Moellmann H et al (2023) Short-Term outcomes of tricuspid Edge-to-Edge repair in clinical practice. J Am Coll Cardiol 82:281–291 37207923 10.1016/j.jacc.2023.05.008

[7] Kodali S, Hahn RT, Makkar R, Makar M, Davidson CJ, Puthumana JJ et al (2023) Transfemoral tricuspid valve replacement and one-year outcomes: the TRISCEND study. Eur Heart J 44:4862–4873 37930776 10.1093/eurheartj/ehad667

[8] Gray WA, Abramson SV, Lim S, Fowler D, Smith RL, Grayburn PA et al (2022) 1-Year outcomes of cardioband tricuspid valve reconstruction system early feasibility study. JACC Cardiovasc Interv 15:1921–1932 36202561 10.1016/j.jcin.2022.07.006

[9] Wild MG, Gloeckler M, Wustmann KB, Erne SA, Grogg H, Huber AT et al (2021) Multimodality imaging for evaluation of bicaval valved stent implantation in severe tricuspid regurgitation. JACC Case Rep 3:1512–1518 34746850 10.1016/j.jaccas.2021.07.009 PMC8551505

[10] Angellotti D, Mattig I, Samim D, Goebel B, Jantsch C, Rubinic B et al (2025) Early outcomes of real-world transcatheter tricuspid valve replacement. JACC Cardiovasc Interv 18(15):1896–1909. 10.1016/j.jcin.2025.06.002 40560107 10.1016/j.jcin.2025.06.002

[11] Hagemeyer D, Merdad A, Sierra LV, Ruberti A, Kargoli F, Bouchat M et al (2024) Clinical characteristics and outcomes of patients screened for transcatheter tricuspid valve replacement: the triact registry. JACC Cardiovasc Interv 17:552–560 38418058 10.1016/j.jcin.2023.12.016

[12] Zoghbi WA, Adams D, Bonow RO, Enriquez-Sarano M, Foster E, Grayburn PA et al (2017) Recommendations for noninvasive evaluation of native valvular regurgitation: A report from the American society of echocardiography developed in collaboration with the society for cardiovascular magnetic resonance. J Am Soc Echocardiogr 30:303–371 28314623 10.1016/j.echo.2017.01.007

[13] Lancellotti P, Pibarot P, Chambers J, La Canna G, Pepi M, Dulgheru R et al (2022) Multi-modality imaging assessment of native valvular regurgitation: an EACVI and ESC council of valvular heart disease position paper. Eur Heart J Cardiovasc Imaging 23:e171–e232 35292799 10.1093/ehjci/jeab253

[14] -Hahn RT, Zamorano JL (2024) Tricuspid regurgitation severity grades: is more always better?? Eur Heart J Cardiovasc Imaging 25(8):1087–1088. 10.1093/ehjci/jeae143 38829783 10.1093/ehjci/jeae143

[15] Barreiro-Perez M, Gonzalez-Ferreiro R, Caneiro-Queija B, Tavares-Silva M, Puga l, Parada-Barcia JA et al (2023) Transcatheter tricuspid valve replacement: illustrative case reports and review of state-of-art. J Clin Med 12(4):1371. 10.3390/jcm12041371 36835907 10.3390/jcm12041371 PMC9967402

[16] Barbieri F, Niehues SM, Feuchtner GM, Skurk C, Landmesser U, Polak-Krasna K et al (2024) Cardiac computed tomography screening for tricuspid transcatheter annuloplasty implantation. Circ Cardiovasc Imaging 17(5):e016292 38708594 10.1161/CIRCIMAGING.123.016292 PMC11111314

[17] Miura M, Alessandrini H, Alkhodair A, Attinger-Toller A, Biasco L, Lurz P et al (2020) Impact of massive or torrential tricuspid regurgitation in patients undergoing transcatheter tricuspid valve intervention. JACC Cardiovasc Interv 13:1999–2009 32912460 10.1016/j.jcin.2020.05.011

[18] Peri Y, Sadeh B, Sherez C, Hochstadt A, Biner S, Aviram G et al (2020) Quantitative assessment of effective regurgitant orifice: impact on risk stratification, and cut-off for severe and torrential tricuspid regurgitation grade. Eur Heart J Cardiovasc Imaging 21:768–776 31642895 10.1093/ehjci/jez267

[19] Samim D, Praz F, Cochard B, Brugger N, Ruberti A, Bartkowiak J et al (2022) Natural history and mid-term prognosis of severe tricuspid regurgitation: A cohort study. Front Cardiovasc Med 9:1026230 36698931 10.3389/fcvm.2022.1026230 PMC9870052

[20] Angellotti D, Franzone A, Brugger N, Reineke D, Esposito G, Praz F (2025) Optimizing the management of tricuspid regurgitation: an update on current treatment strategies and perspectives. Expert Rev Cardiovasc Ther 23(4):131–139 10.1080/14779072.2025.2488869 40177965

[21] Hausleiter J, Stolz L, Lurz P, Rudolph V, Hahn R, Estévez-Loureiro R, Davidson C, Zahr F, Kodali S, Makkar R, Cheung A, Lopes RD, Maisano F, Fam N, Latib A, Windecker S, Praz F (2025) Transcatheter tricuspid valve replacement. J Am Coll Cardiol 85(3):265–291 39580719 10.1016/j.jacc.2024.10.071

[22] Muraru D, Addetia K, Guta A, RC Ochoa-Jimenez R, Genovese D, Veronesi F et al (2021) Right atrial volume is a major determinant of tricuspid annulus area in functional tricuspid regurgitation: a three-dimensional echocardiographic study. Eur Heart J Cardiovasc Imaging 22(6):660–669 Erratum in: Eur Heart J Cardiovasc Imaging. 2021;22(6):669 33387441 10.1093/ehjci/jeaa286

[23] Utsunomiya H, Yoshida J, Izumi K, Ueda Y, Nakano Y, Shiota T et al (2022) Predominant posterior annular dilatation is associated with vena contracta morphology in atrial functional tricuspid regurgitation. J Am Soc Echocardiogr 35(6):588–599 35091070 10.1016/j.echo.2022.01.009

[24] Hahn RT, Lawlor MK, Davidson CJ, Badhwar V, Sannino A, Spitzer E et al (2023) TVARC steering committee. tricuspid valve academic research consortium definitions for tricuspid regurgitation and trial endpoints. J Am Coll Cardiol 82(17):1711–1735 37804294 10.1016/j.jacc.2023.08.008

[25] Muraru D, Badano LP, Hahn RT, Lang RM, Delgado V, Wunderlich NC et al (2024) Atrial secondary tricuspid regurgitation: pathophysiology, definition, diagnosis, and treatment. Eur Heart J 45(11):895–911 38441886 10.1093/eurheartj/ehae088 PMC11095052

[26] Praz F, Khalique OK, Dos Reis Macedo LG, Pulerwitz TC, Jantz J, Wu IY et al (2018) Comparison between three-dimensional echocardiography and computed tomography for comprehensive tricuspid annulus and valve assessment in severe tricuspid regurgitation: implications for tricuspid regurgitation grading and transcatheter therapies. J Am Soc Echocardiogr 31(11):1190-1202e3 30269909 10.1016/j.echo.2018.07.007

[27] Kawata T, Daimon M, Nakanishi K, Kimura K, Sawada N, Nakao T et al (2022) Factors influencing inferior vena cava diameter and its respiratory variation: simultaneous comparison with hemodynamic data. J Cardiol 79(5):642–647 34895983 10.1016/j.jjcc.2021.11.022

[28] van Rosendael PJ, Kamperidis V, Kong WK, van Rosendael AR, van der Kley F, Ajmone Marsana N et al (2017) Computed tomography for planning transcatheter tricuspid valve therapy. Eur Heart J 38:665–674 27807057 10.1093/eurheartj/ehw499

[29] Angellotti D, Bartkowiak J, Samim D, Hunziker L, Brugger N, Windecker S et al (2025) Acute haemodynamic changes following transcatheter tricuspid valve replacement. Eur J Heart Fail. 10.1002/ejhf.3781 40880126 10.1002/ejhf.3781

