# Supplementary material for: CT assessment of right heart anatomy across tricuspid regurgitation severity grades: implications for transcatheter interventions
Source: Int J Cardiovasc Imaging. 2025 Sep 3;41(10):2013–23. doi: 10.1007/s10554-025-03505-8 (PMC12491369; doi:10.1007/s10554-025-03505-8)

**Supplement Appendix**

**Multimodality Imaging Assessment in Patients with Significant Tricuspid Regurgitation: Implications for Transcatheter Treatment**

Table of contents

[Computed Tomography Anatomical parameters collected: all measurements were performed in mid diastole and repeated in systole. 2](#_Toc162909040)

Clinical and anatomical Criteria used to assess eligibility for transcatheter tricuspid valve interventions……………………………………………………………………………………………………….…3

Table S1. Pearson correlation and linear regression between vena contracta at echocardiography and CT-derived measurements……………………………………………………………………..…………………………………4

Table S2. Interobserver Variability analysis with intraclass correlation coefficient (ICC) for CT Measurements in ten patients……………………………………………………………………………………………………………….6

Figure S1 Pearson correlation matrix between echocardiographic quantitative TR grading parameters (Vena contracta and regurgitant volume) and the main CT dimensions. Tricuspid annulus dimensions are derived from CT.……………………………………………………………………………………………………………………7

# List of Computed Tomography Anatomical Parameters Analyzed: all measurements were performed in diastole and systole.

1. Tricuspid Annulus Area (cm^2^)
2. Tricuspid Annulus Maximum Diameter (mm)
3. Tricuspid Annulus Minimum Diameter (mm)
4. Tricuspid Annulus perimeter (mm)
5. Tricuspid Annulus perimeter projected (mm)
6. Minimum distance between tricuspid annulus and right coronary artery (mm)
7. 4-chamber view annulus diameter (mm)
8. 4-chamber view maximum Right Ventricle length (mm)
9. 4-chamber view maximum Right Atrium length (mm)
10. 4-chamber view Right Atrium diameter perpendicular to maximum length (mm)
11. 4-chamber view Right Ventricle basal diameter (mm)
12. 2-chamber view tricuspid annulus diameter (mm)
13. 2-chamber view maximum Right Ventricle length (mm)
14. 2-chamber view maximum Right Atrium length (mm)
15. 2-chamber view Right Atrium diameter perpendicular to maximum length (mm)
16. 2-chamber view Right Ventricle basal diameter (mm)
17. 3-chamber view annulus diameter (mm)
18. 3-chamber view maximum Right Ventricle length (mm)
19. 3-chamber view maximum Right Atrium length (mm)
20. Distance between Inferior cava vein and superior cava vein (mm)
21. Superior cava vein area (mm^2^)
22. Superior cava vein Perimeter (mm)
23. Superior cava vein minimum diameter (mm)
24. Superior cava vein maximum diameter (mm)
25. Superior cava vein area at two cm from the ostium (mm^2^)
26. Superior cava vein area at two cm from the ostium perimeter (mm)
27. Superior cava vein area at two cm from the ostium minimum diameter (mm)
28. Superior cava vein area at two cm from the ostium maximum diameter (mm)
29. Inferior cava vein area (mm^2^)
30. Inferior cava vein Perimeter (mm)
31. Inferior cava vein minimum diameter (mm)
32. Inferior cava vein maximum diameter (mm)
33. Distance between inferior cava vein and hepatic veins
34. Inferior cava vein at the conjunction with hepatic veins area (mm^2^)
35. Inferior cava vein at the conjunction with hepatic veins perimeter (mm)
36. Inferior cava vein at the conjunction with hepatic veins minimum diameter (mm)
37. Inferior cava vein at the conjunction with hepatic veins maximum diameter (mm)

**Computed Tomography Criteria used to determine patient eligibility for each transcatheter tricuspid valve intervention**

1. **EVOQUE tricuspid valve replacement System**
   1. CT criteria: TV annulus projected perimeter at mid-diastole between 114 and 180 mm, TV annulus perimeter derived diameter between 36.5 and 57.5 mm
   2. Clinical and Echocardiographic criteria: TAPSE > 13 mm, LVEF > 25% and sPAP < 70 mmHg by TTE
2. **Cardioband Tricuspid System**
   1. CT criteria: TA perimeter from aorta to coronary sinus between 73 and 120 mm; RCA proximity to the anchors > 6.90 mm and RA size >89 mm
   2. Clinical and Echocardiographic criteria: estimated sPAP <65 mmHg by TTE
3. **Cardiovalve transfemoral valve replacement system**
   1. CT criteria: TA diameter at mid-diastole ranging between 45 and 60 mm
   2. Clinical and Echocardiographic criteria: TAPSE >13 mm by TTE
4. **Tricvalve® transcatheter bicaval valve system**
   1. CT criteria:
5. Superior cava vein (SVC) valve: diameter of SVC at level of top of Pulmonary Artery (19-34 mm), at level of middle of Pulmonary Artery (22-34 mm), diameter of confluence larger than 14 mm, lenght of middle PA larger than 35 mm, lenght to SVC-RA larger than 50 mm.
6. Inferior cava vein (IVC) valve: IVC-RA junction (24-35 mm), IVC top HV (24-35 mm), Length IVC/RA junction – Hepatic veins Larger than 10mm, IVC just below HV (21-43 mm), IVC 5cm below RA junction (21-43 mm)
   1. Clinical and Echocardiographic criteria: TAPSE > 13 mm, sPAP < 65 mmHg as assessed by TTE
7. **LuX-Valve Plus tricuspid valve replacement System**
   1. CT criteria: TA diameter at mid-diastole ranged between 35 and 75 mm
   2. Clinical and Echocardiographic criteria: TAPSE > 10 mm, sPAP < 65 mmHg as assessed by TTE

**Table S1. Pearson correlation and linear regression between vena contracta at echocardiography and CT-derived measurements.**

| CT measurements | TTE/TEE Vena contracta Pearson Coefficient *r* | | Correlation strength (Strong: *r* > 0.60; Moderate: *r* = 0.40-0.59; Poor: *r* < 0.40) | | p value | | Standardized Coefficient Beta | | p value | |
| --- | --- | --- | --- | --- | --- | --- | --- | --- | --- | --- |
|  | Systole | Diastole | Systole | Diastole | Systole | Diastole | Systole | Diastole | Systole | Diastole |
| Annulus area, cm^2^ | 0.441 | 0.407 | moderate | moderate | <0.001 | <0.001 | 2.080 | 1.405 | 0.132 | 0.064 |
| Annulus max. diameter (mm) | 0.417 | 0.391 | moderate | poor | <0.001 | <0.001 | -0.134 | 0.071 | 0.631 | 0.895 |
| Annulus min. diameter (perpendicular to max. diam.) (mm) | 0.401 | 0.349 | moderate | poor | <0.001 | 0.002 | -0.547 | -0.669 | 0.202 | 0.073 |
| Annulus perimeter (mm) | 0.414 | 0.363 | moderate | poor | <0.001 | 0.001 | 0.463 | -0.272 | 0.649 | 0.654 |
| Annulus perimeter projected (mm) | 0.439 | 0.378 | moderate | poor | <0.001 | 0.001 | -0.140 | 0.019 | 0.934 | 0.967 |
| RCA Distance min. from TV annulus (mm) | 0.143 | 0.350 | poor | poor | 0.116 | 0.001 | -0.034 | -0.110 | 0.799 | 0.504 |
| 4CH Annulus diameter (mm) | 0.350 | 0.312 | poor | poor | 0.001 | 0.004 | 0,138 | 0.352 | 0.633 | 0.260 |
| 4CH Max. RV length (mm) | 0.235 | 0.279 | poor | poor | 0.024 | 0.010 | -0.080 | -0.050 | 0.722 | 0.810 |
| 4CH Max. RA length (mm) | 0.450 | 0.445 | moderate | moderate | <0.001 | <0.001 | -0.264 | 0.479 | 0.540 | 0.256 |
| 4CH RA diameter perpendicular to max. length (mm) | 0.369 | 0.085 | poor | poor | 0.001 | 0.242 | 0.137 | 0.042 | 0.553 | 0.734 |
| 4CH RV base (mm) | 0.188 | 0.318 | poor | poor | 0.058 | 0.004 | -0.211 | -0.171 | 0.406 | 0.448 |
| 2CH Annulus diameter (mm) | 0.226 | 0.122 | poor | poor | 0.029 | 0.157 | -0.910 | -0.725 | 0.005 | 0.025 |
| 2CH Max. RV length (mm) | 0.061 | 0.094 | poor | poor | 0.308 | 0.220 | 0.103 | 0.115 | 0.631 | 0.529 |
| 2CH Max RA length (mm) | 0.215 | 0.157 | poor | poor | 0.036 | 0.097 | -0.414 | -0.302 | 0.140 | 0.311 |
| 2CH RA diameter perpendicular to max. length (mm) | 0.465 | 0.503 | moderate | moderate | <0.001 | <0.001 | -0.061 | 0.145 | 0.779 | 0.580 |
| 2CH RV base (mm) | 0.190 | 0.295 | poor | poor | 0.056 | 0.007 | -0.065 | 0.067 | 0.753 | 0.770 |
| 3CH Annulus diameter (mm) | 0.232 | 0.264 | poor | poor | 0.026 | 0.014 | -0.183 | -0.151 | 0.523 | 0.572 |
| 3CH Max. RV length (mm) | 0.235 | 0.295 | poor | poor | 0.024 | 0.007 | -0.007 | -0.048 | 0.975 | 0.813 |
| 3CH Max. RA length (mm) | 0.448 | 0.426 | moderate | moderate | <0.001 | <0.001 | 0.518 | -0.158 | 0.284 | 0.747 |
| Distance IVC-SVC (mm) | 0.236 | 0.311 | poor | poor | 0.024 | 0.004 | -0.564 | -0.179 | 0.021 | 0.464 |
| SVC Area (mm^2^) | 0.416 | 0.374 | moderate | poor | <0.001 | 0.001 | 0.193 | 0.007 | 0.771 | 0.995 |
| SVC Perimeter (mm) | 0.334 | 0.373 | poor | poor | 0.002 | 0.001 | 0.333 | 0.220 | 0.338 | 0.877 |
| SVC Min. diameter (mm) | 0.394 | 0.362 | poor | poor | <0.001 | 0.001 | -0.081 | 0.034 | 0.873 | 0.960 |
| SVC Max. diameter (mm) | 0.022 | 0.387 | poor | poor | 0.429 | <0.001 | -0.288 | -0.228 | 0.038 | 0.740 |
| 2cmSVC Area (mm^2^) | 0.355 | 0.398 | poor | poor | 0.001 | <0.001 | -0.357 | 0.454 | 0.382 | 0.734 |
| 2cmSVC Perimeter (mm) | 0.386 | 0.404 | poor | moderate | <0.001 | <0.001 | 0.126 | 1.848 | 0.653 | 0.209 |
| 2cmSVC Min. diameter (mm) | 0.412 | 0.330 | moderate | poor | <0.001 | 0.003 | -0.495 | 0.117 | 0.288 | 0.870 |
| 2cmSVC Max. diameter (mm) | 0.420 | 0.457 | moderate | moderate | <0.001 | <0.001 | 0.511 | 1.630 | 0.152 | 0.057 |
| IVC Area (mm^2^) | 0.237 | 0.167 | poor | poor | 0.023 | 0.083 | 2.585 | 0.842 | 0.024 | 0.401 |
| IVC Perimeter (mm) | 0.212 | 0.194 | poor | poor | 0.038 | 0.054 | -0.109 | 0.640 | 0.775 | 0.543 |
| IVC Min. diameter (mm) | 0.198 | 0.121 | poor | poor | 0.049 | 0.160 | -1.391 | -0.629 | 0.047 | 0.310 |
| IVC Max. diameter (mm) | 0.216 | 0.208 | poor | poor | 0.035 | 0.042 | -1.386 | -0.900 | 0.036 | 0.353 |
| IVC Distance to the liver vein(mm) | -0.152 | -0.162 | poor | poor | 0.103 | 0.091 | -0.193 | -0.036 | 0.248 | 0.778 |
| IVCaHV Area (mm^2^) | 0.367 | 0.303 | poor | poor | 0.001 | 0.005 | -2.174 | 0.444 | 0.085 | 0.698 |
| IVCaHV Perimeter (mm) | 0.380 | 0.293 | poor | poor | 0.001 | 0.007 | 0.867 | -1.019 | 0.304 | 0.476 |
| IVCaHV Min. diameter (mm) | 0.401 | 0.338 | moderate | poor | <0.001 | 0.002 | 1.095 | 0.493 | 0.014 | 0.182 |
| IVCaHV Max. diameter (mm) | 0.262 | 0.238 | poor | poor | 0.014 | 0.023 | 0.553 | 0.220 | 0.188 | 0.737 |

**Table S2. Interobserver Variability analysis with intraclass correlation coefficient (ICC) for CT Measurements in 10 randomly chosen patients**

| **Patient Number** | **Observer** | **Tricuspid Annulus Area, cm2** | **Tricuspid Annulus perimeter, mm** | **D SVC Area, mm2** | **D IVC Area, mm2** |
| --- | --- | --- | --- | --- | --- |
| **1** | A.S | 12.5 | 133.4 | 404.8 | 351.7 |
|  | D.A | 11.8 | 128.4 | 376 | 348 |
| **2** | A.S | 12.7 | 136.1 | 341.2 | 435.1 |
|  | D.A | 11 | 128.4 | 360 | 422.2 |
| **3** | A.S | 18.3 | 156.4 | 694.3 | 881.9 |
|  | D.A | 16.7 | 154.8 | 686.2 | 869.8 |
| **4** | A.S | 15.8 | 148.7 | 489.8 | 718.6 |
|  | D.A | 14.7 | 146.9 | 488.4 | 708.9 |
| **5** | A.S | 25.3 | 190.2 | 1132.5 | 1390.8 |
|  | D.A | 24.1 | 186.9 | 999.8 | 992.4 |
| **6** | A.S | 12.3 | 128.1 | 400.4 | 550.1 |
|  | D.A | 11.4 | 124 | 433.5 | 582.8 |
| **7** | A.S | 12.6 | 146.3 | 518.8 | 189.7 |
|  | D.A | 11.8 | 140.8 | 495.5 | 200 |
| **8** | A.S | 17.8 | 154.9 | 834.8 | 1296.3 |
|  | D.A | 16.9 | 152.6 | 832.5 | 1289.2 |
| **9** | A.S | 20.9 | 167.6 | 1102.1 | 979 |
|  | D.A | 19.7 | 152.5 | 1188.5 | 999.2 |
| **10** | A.S | 10.8 | 122.9 | 830.9 | 748.3 |
|  | D.A | 10.6 | 122.7 | 824.4 | 771.5 |
| **ICC** |  | **0.949** | **0.931** | **0.867** | **0.835** |

**Figure S1**. Pearson correlation matrix between echocardiographic quantitative TR grading parameters (Vena contracta and regurgitant volume) and the main CT dimensions. Tricuspid annulus dimensions are derived from CT.
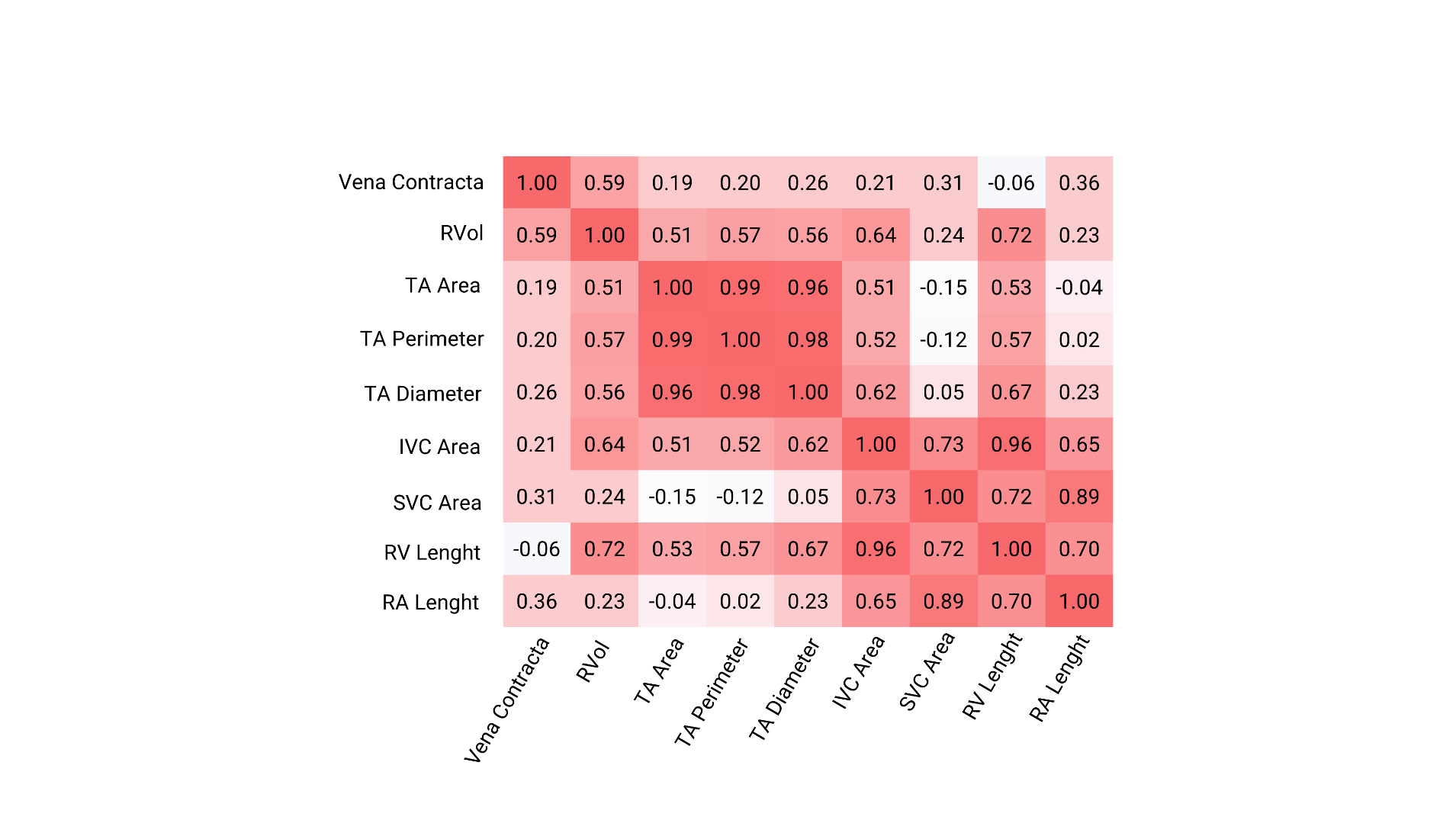

Supplement: Supplementary file 1 — Supplementary Material 1 [file 10554_2025_3505_MOESM1_ESM.docx]
